# Early Serum Free Light Chain Response in Newly Diagnosed Multiple Myeloma: Documented Earlier Than MRD and Complementary in Prognosis

**DOI:** 10.1002/hon.70234

**Published:** 2026-08-04

**Authors:** Yubiao Pan, Yue Wang, Yaqin Xiong, Panpan Li, Chenqi Yu, Peng Liu

**Affiliations:** ^1^ Department of Hematology Zhongshan Hospital Fudan University Shanghai China

**Keywords:** induction therapy, minimal residual disease, multiple myeloma, prognostic biomarker, serum free light chains

## Abstract

Minimal residual disease (MRD) is the standard for deep response assessment in multiple myeloma but requires bone marrow sampling. Whether early serum free light chain (sFLC) response provides independent prognostic information in real‐world, predominantly non‐transplant patients remains unclear. We retrospectively analyzed 701 patients with newly diagnosed multiple myeloma treated at a single Chinese center (2015–2021). sFLC ratio normalization (IMWG range 0.26–1.65) during the first four induction cycles was assessed using a 4‐month landmark to mitigate immortal time bias. Multivariable Cox models adjusted for R‐ISS and age, with prespecified sensitivity analyses and direct comparison with established markers and MRD. Among 701 patients (median age 64 years; ASCT 12.6%), 433 (61.8%) were classified as FLC‐normalized during C1‐C4. FLC non‐normalization was associated with inferior PFS (HR 2.10, 95% CI 1.60–2.76) and OS (HR 1.96, 95% CI 1.32–2.90) after adjustment for R‐ISS stage and age. The association persisted in baseline‐abnormal patients and after MRD adjustment. Adding sFLC status produced a modest improvement in model discrimination, supporting a complementary rather than stand‐alone prognostic role. sFLC normalization was documented earlier than MRD negativity, a pattern that persisted in a paired‐visit‐restricted sensitivity analysis, although timing comparisons remain subject to retrospective assessment schedules. Early sFLC response was associated with outcomes in newly diagnosed multiple myeloma and provided complementary, incremental prognostic information beyond established risk factors and MRD. These findings support further prospective evaluation of sFLC normalization as a pragmatic blood‐based marker for early risk stratification.

## 1 Introduction

Multiple myeloma (MM) accounts for approximately 10% of hematologic malignancies [1, 2]. Despite proteasome inhibitors (PIs), immunomodulatory drugs (IMiDs), and autologous stem cell transplantation (ASCT), most patients remain incurable, with heterogeneous outcomes driven by tumor biology and treatment response depth.

Early identification of patients at high risk of treatment failure is critical for clinical decision‐making. The International Myeloma Working Group (IMWG) recommends response assessment after approximately four cycles of induction therapy to guide subsequent therapeutic strategies including ASCT, consolidation, and maintenance [3, 4, 5, 6]. Established prognostic factors, including R‐ISS [7], its second‐generation update [8], and FISH‐detected cytogenetic abnormalities provide risk stratification at diagnosis but do not capture dynamic treatment response. Response depth assessment by IMWG criteria and minimal residual disease (MRD) testing offer real‐time information but require invasive bone marrow sampling [9, 10]. Although MRD has emerged as the most powerful response marker, its routine clinical use is constrained by several practical limitations: it is performed only at predefined milestones rather than continuously, may miss focal residual disease due to single‐site sampling, and depends on platforms not uniformly available outside specialized centers. These constraints have motivated the search for blood‐based markers that can be monitored more frequently and complement marrow‐based MRD.

Serum free light chains (sFLC) offer a complementary, non‐invasive approach to response monitoring. Unlike serum M‐protein, sFLC are rapidly cleared by the kidneys (half‐life 2–6 hours), providing real‐time information on tumor activity [11]. sFLC ratio normalization during treatment has been proposed as an early biomarker of deep response [12, 13], preceding changes in conventional immunofixation [14, 15]. sFLC dynamics also characterize distinct relapse patterns, including FLC escape [16], and serial sFLC monitoring is informative even in patients with measurable M‐protein [17].

Several studies have evaluated sFLC normalization as a prognostic marker. Iwama et al. reported it as an independent predictor of PFS and OS in 126 Japanese patients (3‐year OS 94% vs. 48%) [12]. Klein et al. (GMMG‐MM5 trial; *n* = 590, PFS HR 0.61) [18] and Tacchetti et al. (*n* = 339, PFS HR 0.46) [13] reported comparable findings. However, both European trial‐based studies were conducted predominantly in transplant‐eligible patients and did not employ landmark analysis to address immortal time bias. Although FLC‐MRD concordance has been described [19], whether FLC provides independent prognostic information beyond MRD, or further stratifies outcomes within MRD‐defined subgroups, has not been established.

In a real‐world Chinese cohort of newly diagnosed MM patients, we evaluated the prognostic impact of FLC ratio normalization during the first four induction cycles. Using a 4‐month landmark to mitigate immortal time bias, we assessed the independent association of FLC non‐normalization with PFS and OS, with prespecified sensitivity analyses restricted to baseline‐abnormal patients and adjusted for MRD status. We additionally evaluated sFLC alongside established baseline markers in a direct comparison.

## 2 Methods

### 2.1 Study Design, Patients, and Definitions

This retrospective cohort study included patients with newly diagnosed MM from the Hematology Department of Zhongshan Hospital, Fudan University between January 2015 and December 2021. Patient data were extracted from an institutional plasma cell disorder (PCD) database comprising 2040 consecutive cases. The study was approved by the Institutional Review Board of Zhongshan Hospital, Fudan University, and written informed consent was obtained from all patients (Approval No. B2017‐031R).

Inclusion criteria were: confirmed MM diagnosis (IMWG 2014), available PFS follow‐up, sFLC measurement during C1–C4, and PFS > 4 months (landmark). Sequential exclusions from 2040 database entries yielded 701 patients in the analysis cohort and 547 in the primary multivariable model (Supporting Information S1: Figure S1).

The FLC ratio was considered normal within the IMWG range of 0.26–1.65. Patients were classified as FLC‐normalized if any sFLC ratio assessment during cycles C1‐C4 was within this range, and as FLC non‐normalized otherwise. Because the normal range is applied regardless of baseline status, the FLC‐normalized group included both treatment‐induced normalizers and patients whose baseline ratio was already within the normal range.

**FIGURE 1:**
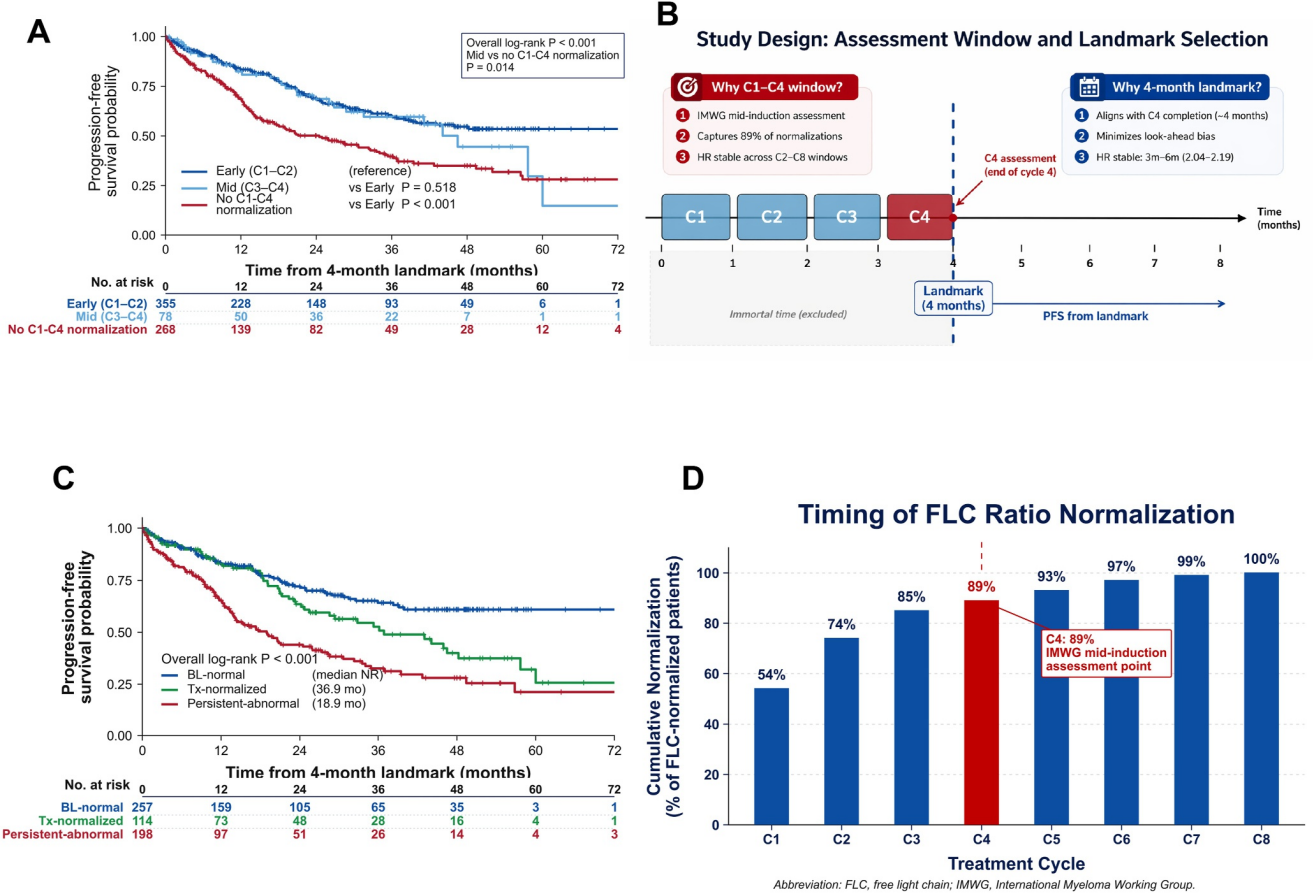
Normalization timing, study design, baseline FLC stratification, and assessment window validation. (A) PFS stratified by timing of first FLC ratio normalization: early (C1–C2, *n* = 355), mid‐course (C3–C4, *n* = 78), and no C1–C4 normalization (*n* = 268). Overall log‐rank *p* < 0.001. Early versus mid‐course, *p* = 0.518; early versus no C1–C4 normalization, *p* < 0.001; mid‐course versus no C1–C4 normalization, *p* = 0.014. Time from 4‐month landmark. (B) Study design schematic illustrating the C1–C4 assessment window and 4‐month landmark selection. The C1–C4 window corresponds to the IMWG‐recommended mid‐induction assessment point, captures 89% of all normalizations, and yields stable HR estimates across alternative window choices. (C) PFS stratified by baseline FLC status and normalization response in 569 patients with evaluable baseline FLC ratios: baseline‐normal (*n* = 257), baseline‐abnormal with treatment‐induced normalization (*n* = 114), and persistent abnormality (*n* = 198). Median PFS: not reached, 36.9, and 18.9 months, respectively (overall *p* < 0.001). (D) Cumulative proportion of the 482 patients who achieved FLC ratio normalization during cycles C1–C8: 54% by C1, 74% by C2, 85% by C3, 89% by C4, and 100% by C8. The C4 time point (red) corresponds to the IMWG mid‐induction assessment point and captures the majority of patients who achieved normalization by C8.

C1–C4 was prespecified as the assessment window, aligning with the IMWG mid‐induction response evaluation point (Figure 1B). Stability across alternative windows is shown in Supporting Information S1: Figure S2.

The primary outcome was PFS, defined clinically as time to disease progression or death from any cause, whichever occurred first. The secondary outcome was overall survival (OS), defined clinically as time to death from any cause. In all landmark analyses, the time scale was time since the 4‐month landmark; events occurring before the landmark were excluded. Disease response and progression were assessed according to IMWG uniform response criteria [20].

### 2.2 Statistical Analysis

A landmark analysis approach was used to mitigate immortal time bias inherent in evaluating early treatment response markers [21, 22]. The landmark time was set at 4 months from diagnosis, corresponding to the expected completion of four 28‐day treatment cycles. Patients with PFS time ≤ 4 months were excluded from the landmark cohort (*n* = 61), including 39 patients with pre‐landmark PFS events and 22 patients whose PFS follow‐up did not extend beyond the landmark. PFS and OS were measured from the 4‐month landmark rather than from diagnosis. For landmark sensitivity analyses, we repeated the adjusted Cox model at 3‐, 4‐, 5‐, and 6‐month landmarks and tabulated, for each landmark definition, the number of patients and PFS events excluded before the landmark, the complete‐case Cox model population, and the adjusted HR with 95% CI.

Continuous variables were reported as median (IQR) and compared using Mann–Whitney *U* test; categorical variables as *n* (%) compared using chi‐squared or Fisher's exact test.

**FIGURE 2:**
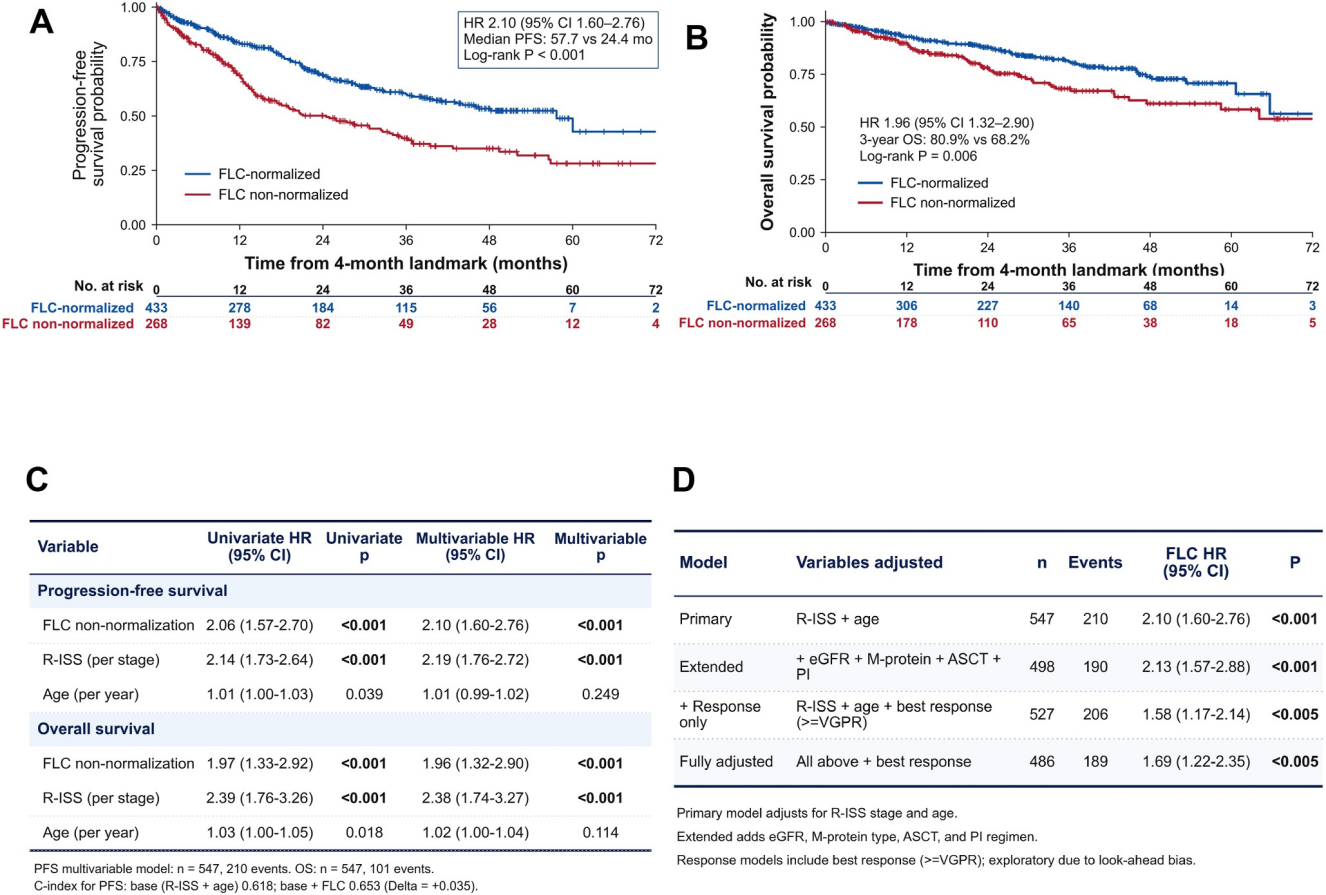
FLC ratio normalization is associated with progression‐free survival and overall survival. (A) PFS and (B) OS stratified by FLC normalization status during C1–C4 (FLC‐normalized, *n* = 433; FLC non‐normalized, *n* = 268). Median PFS: 57.7 versus 24.4 months (log‐rank *p* < 0.001). Three‐year OS: 80.9% versus 68.2% (*p* = 0.006). Time measured from the 4‐month landmark. (C) Univariable and multivariable Cox proportional hazards analysis for PFS and OS, adjusting for R‐ISS stage and age (*n* = 547, 210 PFS events, 101 OS events). Model discrimination (C‐index) for PFS: base model (R‐ISS + age) 0.618; base + FLC 0.653 (Δ = +0.035). (D) Extended multivariable models for PFS showing stability of FLC non‐normalization HR across progressively adjusted models: primary (R‐ISS + age, HR 2.10), extended (+eGFR, M‐protein, ASCT, PI, HR 2.13), response‐adjusted (+ best response ≥ VGPR, HR 1.58), and fully adjusted (HR 1.69). All models use PFS as the outcome with a 4‐month landmark.

Kaplan–Meier curves were compared using the log‐rank test. Univariable and multivariable Cox models estimated HRs with 95% CIs. The primary model included FLC normalization status (FLC‐normalized vs. FLC non‐normalized), R‐ISS (continuous ordinal), and age. FISH cytogenetics, ASCT, eGFR, baseline M‐protein, and first‐line regimen were evaluated in extended sensitivity models (Figure 2D). Model discrimination was assessed using Harrell's C‐index; the proportional hazards assumption was not violated.

Prespecified subgroup analyses were stratified by age, sex, ISS, R‐ISS, cytogenetic risk, ASCT status, and first‐line regimen, with multiplicative interaction terms. Timing of normalization (early C1–C2 vs. mid‐course C3–C4) was also assessed. All interaction *p* values are uncorrected and hypothesis‐generating.

MRD was assessed by next‐generation flow cytometry (NGF; two‐tube, eight‐color panel, sensitivity ≥ 10^−5^), as previously described [23]. MRD assessments were available for 627/701 patients (89.4%), with a median of 2 per patient (mostly C2 and C4). Patients were classified as “ever MRD‐negative” if they achieved negativity at any C1–C4 assessment, paralleling the “C1–C4 FLC‐normalized” definition. MRD was included as an additional covariate in an exploratory Cox model (*n* = 495).

Sensitivity analyses included: (S1) no landmark; (S2) expanded window (C1–C8); (S3) exclusion of ASCT‐missing; (S4) descriptive comparison of late (C5–C8) normalizers with patients with no normalization through C8; (S5) restriction to baseline FLC‐abnormal patients; and (S6) adjustment for PI‐based therapy. An extended multivariable model added eGFR, baseline M‐protein, ASCT, PI therapy, and best response (≥ VGPR) to R‐ISS and age (Figure 2D).

To address monitoring‐density bias, we compared sFLC measurement counts between groups and performed Cox models restricting to patients with ≥ 2 measurements or an evaluable C4 measurement, and adjusting for measurement count. Exploratory FLC‐MRD comparisons included time‐to‐event analysis, multivariable Cox models, and sensitivity analyses under alternative MRD definitions. To further address potential measurement‐frequency bias between sFLC and MRD assessments, we performed a paired‐visit‐restricted sensitivity analysis in patients with baseline‐abnormal FLC and available paired sFLC/MRD visits. In this analysis, both FLC normalization and MRD negativity were evaluated only at cycle visits with concurrent sFLC ratio and MRD results. Patients without documented normalization or MRD negativity were censored at the last paired visit.

Statistical analyses were performed using Python 3.10 (lifelines 0.27, scipy 1.11). A two‐sided *p* value < 0.05 was considered statistically significant.

## 3 Results

### 3.1 Patient Characteristics

A total of 701 patients met the inclusion criteria (Supporting Information S1: Figure S1). The median age was 64 years (IQR 57–71), and 440 (62.8%) were male. ISS stage III was present in 299 (42.7%); among 547 with R‐ISS, stage II was most common (319, 58.3%). High‐risk FISH was identified in 152/608 (25.0%). First‐line therapy was PI+IMiD‐based in 339 (48.4%), PI‐only in 253 (36.1%); 88 (12.6%) underwent ASCT. Baseline characteristics by FLC group are summarized in Table 1.

**TABLE 1:** Baseline characteristics.

Variable	Total (*n* = 701)	FLC‐normalized (*n* = 433)	FLC non‐normalized (*n* = 268)	*p* value
Age, median (IQR)	64.0 (57.0–71.0)	65.0 (57.0–71.0)	64.0 (57.0–70.0)	0.720
Male, *n* (%)	440 (62.8)	267 (61.7)	173 (64.6)	0.491
ISS stage, *n* (%)				0.191
1	200 (28.5)	134 (30.9)	66 (24.6)	
2	201 (28.7)	118 (27.3)	83 (31.0)	
3	299 (42.7)	181 (41.8)	118 (44.0)	
R‐ISS stage, *n* (%)				0.278
1	125 (22.9)	85 (24.9)	40 (19.4)	
2	319 (58.3)	191 (56.0)	128 (62.1)	
3	103 (18.8)	65 (19.1)	38 (18.4)	
High‐risk FISH, *n* (%)	152 (25.0)	94 (24.9)	58 (25.2)	1.000
Bone marrow plasma cells (%), median (IQR)	12.0 (4.5–29.5)	8.5 (3.2–24.5)	17.8 (7.0–33.9)	**< 0.001**
Hemoglobin (g/L), median (IQR)	107.0 (87.0–125.0)	110.0 (90.2–127.0)	103.0 (82.0–120.8)	**< 0.001**
Corrected calcium (mmol/L), median (IQR)	2.3 (2.1–2.4)	2.3 (2.1–2.4)	2.2 (2.1–2.4)	0.845
Beta‐2 microglobulin, median (IQR)	4.4 (2.9–7.4)	4.1 (2.7–7.1)	4.7 (3.2–7.6)	**0.018**
Albumin (g/L), median (IQR)	37.0 (32.0–42.0)	37.0 (32.0–42.0)	38.0 (32.0–42.0)	0.483
eGFR (CKD‐EPI), median (IQR)	74.0 (48.0–93.0)	75.0 (49.0–92.0)	70.0 (45.0–94.0)	0.631
LDH (U/L), median (IQR)	169.0 (143.0–214.0)	169.0 (143.0–211.0)	173.0 (143.0–216.0)	0.905
Baseline M‐protein, median (IQR)	4.2 (0.2–12.1)	3.5 (0.2–10.1)	5.9 (0.2–17.9)	**0.006**
Baseline FLC ratio abnormal, *n* (%)	312 (54.8)	114 (30.7)	198 (100.0)	**< 0.001**
Immunoglobulin subtype, *n* (%)				**0.023**
IgG	372 (53.1)	231 (53.5)	141 (52.6)	
IgA	178 (25.4)	123 (28.5)	55 (20.5)	
IgD	6 (0.9)	4 (0.9)	2 (0.7)	
Light‐chain only	107 (15.3)	53 (12.3)	54 (20.1)	
Negative/Non‐secretory	37 (5.3)	21 (4.9)	16 (6.0)	
ASCT received, *n* (%)	88 (12.6)	51 (11.8)	37 (13.8)	0.452
Best response ≥ VGPR, *n* (%)	454/671 (67.7)	353/428 (82.5)	101/243 (41.6)	**< 0.001**
First‐line treatment, *n* (%)				0.773
PI+IMiD	339 (48.4)	206 (47.6)	133 (49.6)	
PI‐only	253 (36.1)	161 (37.2)	92 (34.3)	
IMiD‐only	24 (3.4)	13 (3.0)	11 (4.1)	
Other	85 (12.1)	53 (12.2)	32 (11.9)	

*Note:* Bold *p* values indicate statistical significance (*p* < 0.05). Continuous variables were compared by Mann–Whitney *U* test; categorical variables by chi‐square or Fisher's exact test.

Abbreviations: ASCT, autologous stem cell transplantation; eGFR, estimated glomerular filtration rate; FISH, fluorescence in situ hybridization; FLC, free light chain; IMiD, immunomodulatory drug; IQR, interquartile range; ISS, International Staging System; LDH, lactate dehydrogenase; PI, proteasome inhibitor; R‐ISS, Revised International Staging System; VGPR, very good partial response.

Of 701 patients, 433 (61.8%) were classified as FLC‐normalized during C1‐C4 and 268 (38.2%) as FLC non‐normalized. FLC non‐normalized patients had significantly higher baseline BM plasma cell infiltration, lower hemoglobin, higher beta‐2 microglobulin, higher serum M‐protein, and a different immunoglobulin subtype distribution, with light‐chain‐only myeloma over‐represented (20.1% vs. 12.3%; Table 1). Among 371 evaluable FLC‐normalized patients, 257 (69.3%) had baseline‐normal FLC ratios. Among the 482 patients who achieved FLC ratio normalization during C1–C8, 89% had normalized by C4 (Figure 1D).

**FIGURE 3:**
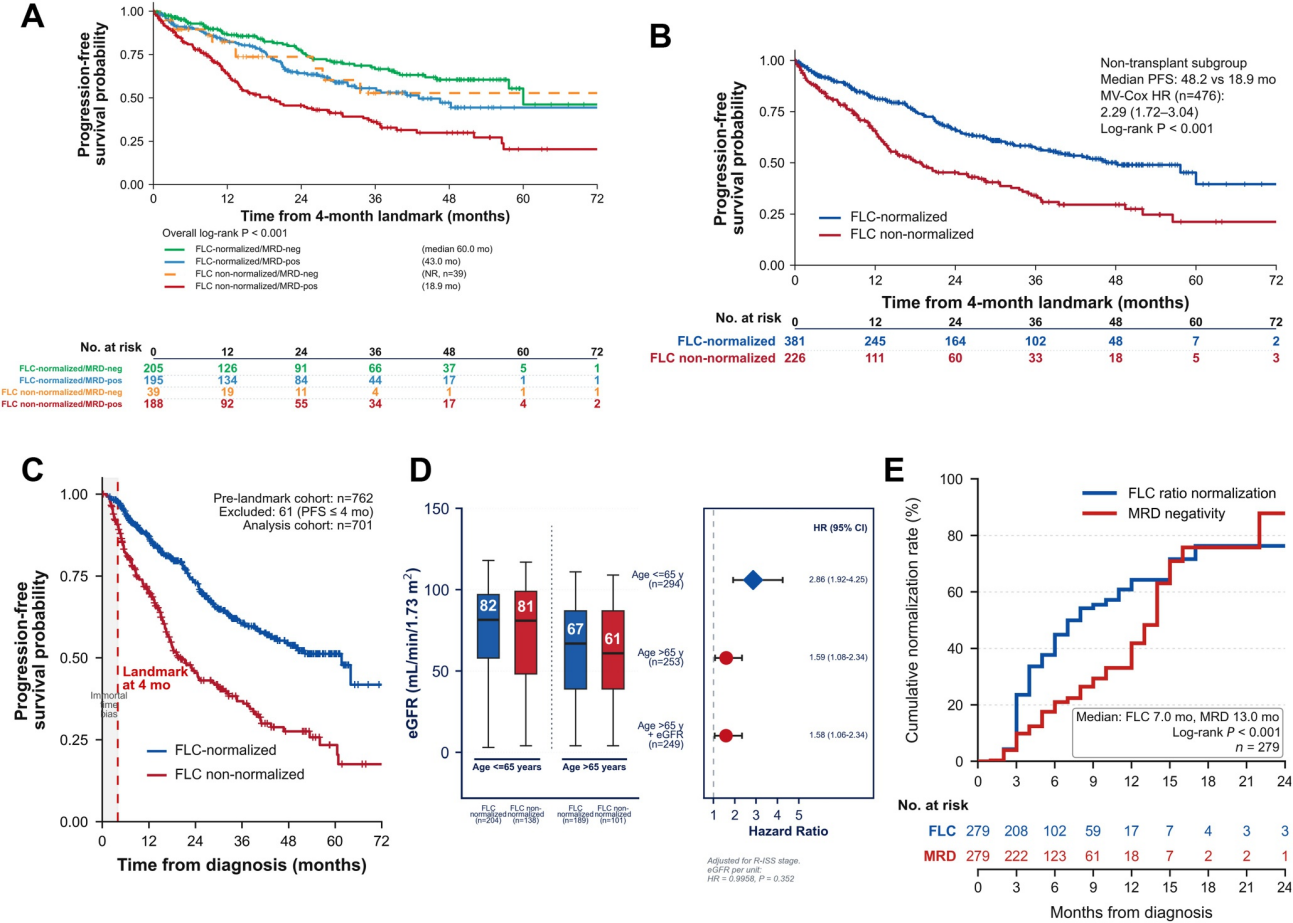
Combined FLC and MRD stratification with key validation analyses. (A) PFS stratified by combined FLC normalization and MRD status during C1–C4 (*n* = 627). Log‐rank *p* < 0.001. Median PFS: FLC‐normalized/MRD‐negative, 60.0 months (*n* = 205); FLC‐normalized/MRD‐positive, 43.0 months (*n* = 195); FLC non‐normalized/MRD‐negative, not reached (*n* = 39; estimate unstable due to small sample size); FLC non‐normalized/MRD‐positive, 18.9 months (*n* = 188). Time from 4‐month landmark. (B) Kaplan–Meier analysis of PFS in the broader non‐transplant population (*n* = 607; FLC‐normalized, *n* = 381; FLC non‐normalized, *n* = 226), independent of R‐ISS availability. Median PFS: 48.2 versus 18.9 months (*p* < 0.001). The corresponding multivariable Cox HR in patients with complete R‐ISS data (*n* = 476) is reported in the main text. Time from 4‐month landmark. (C) Landmark analysis design. KM curves from diagnosis for all 762 patients with FLC data (pre‐landmark); 61 patients with PFS ≤ 4 months were excluded (shaded zone). After landmark exclusion: *n* = 701 analysis cohort. (D) Renal function and age‐stratified FLC prognostic effect. Left: baseline eGFR by age group and FLC status. Right: HRs for FLC non‐normalization from age‐stratified multivariable Cox models. The primary age‐stratified analysis (without eGFR adjustment, *n* = 547) yielded HR 2.86 in patients ≤ 65 years (*n* = 294) and HR 1.59 in patients > 65 years (*n* = 253). In a sensitivity analysis restricted to 541 patients with available eGFR data, the corresponding HRs were 2.78 (≤ 65, *n* = 290) and 1.63 (> 65, *n* = 251); additional adjustment for eGFR yielded HR 1.58 in patients > 65 years (*n* = 251), with eGFR itself not significant (HR 0.9958, *p* = 0.352). (E) Cumulative incidence of FLC ratio normalization and MRD negativity from diagnosis in 279 patients with baseline‐abnormal FLC and at least one MRD assessment. Median time to event: 7.0 months for FLC versus 13.0 months for MRD (log‐rank *p* < 0.001). Cumulative rates at 3/6/12 months: 23.6%/44.9%/64.3% for FLC versus 9.9%/21.0%/41.8% for MRD. The timing comparison reflects differential measurement frequency (median 4 FLC vs. 2 MRD assessments during C1–C4). Time axis represents months from diagnosis (in contrast to other panels in this Figure, which use the 4‐month landmark).

Before application of the 4‐month landmark, 762 patients had C1‐C4 sFLC data and PFS follow‐up. Among them, 61 patients had PFS time ≤ 4 months and were excluded from the landmark cohort (Figure 3C). This group included 39 patients with pre‐landmark PFS events and 22 patients whose PFS follow‐up did not extend beyond 4 months. Compared with the 701 patients included in the landmark cohort, excluded patients showed a higher‐risk profile, with higher proportions of ISS stage III disease, R‐ISS stage III disease, baseline FLC ratio abnormality, and C1‐C4 FLC non‐normalization (Table S5). C1‐C4 FLC non‐normalization was observed in 42/61 excluded patients (68.9%) compared with 268/701 included patients (38.2%).

### 3.2 Survival Outcomes

**FIGURE 4:**
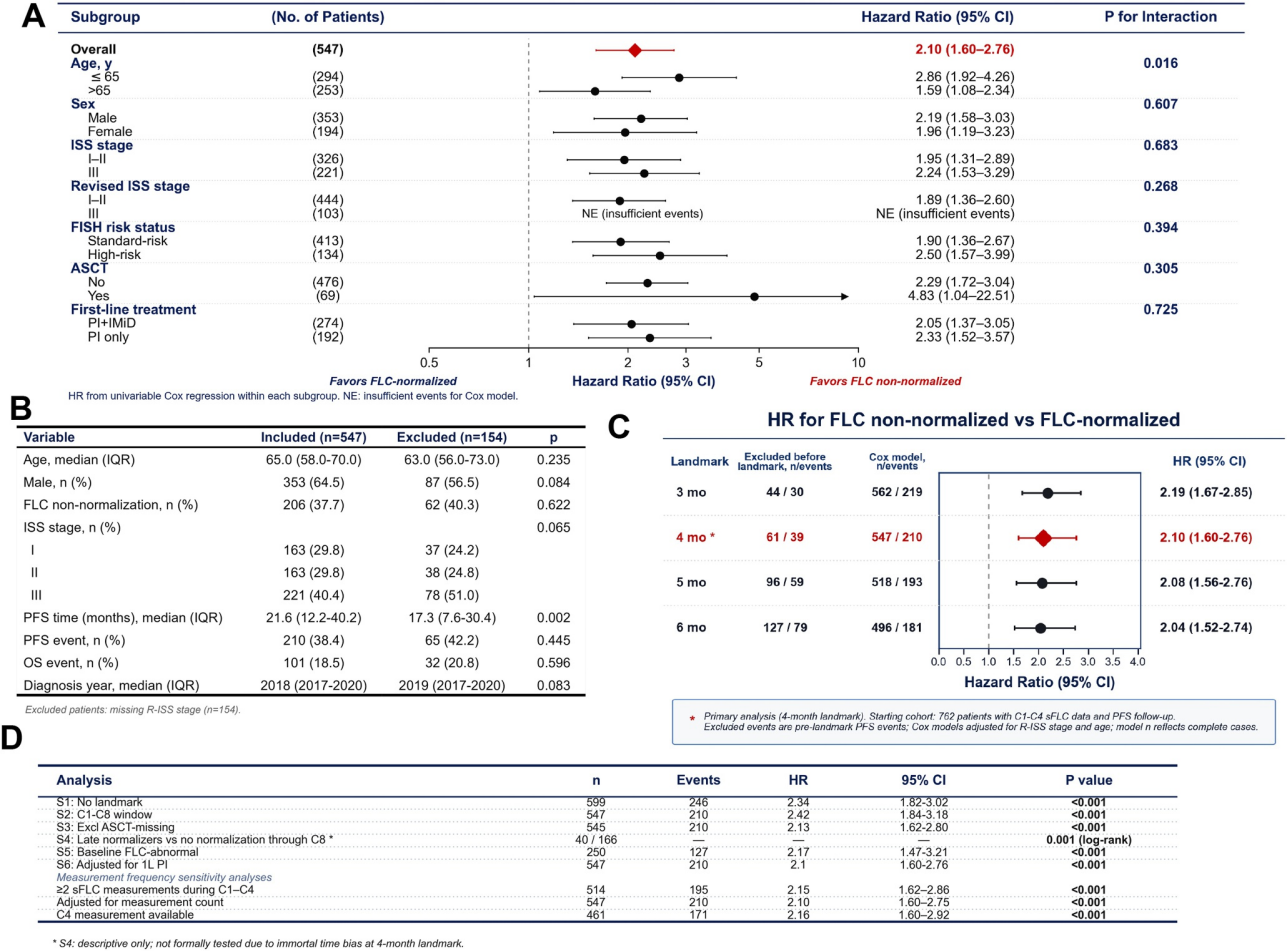
Subgroup consistency and sensitivity analyses. (A) Forest plot of hazard ratios (95% CIs) for FLC non‐normalization across prespecified subgroups. Overall HR 2.10 (1.60–2.76), *n* = 547. Subgroup HRs ranged from 1.59 to 4.83. For the R‐ISS subgroups, multivariable models were adjusted for age only (R‐ISS itself is constant within each subgroup); in the R‐ISS III subgroup (*n* = 103, 60 events), FLC non‐normalization remained associated with inferior PFS (HR 2.59, 95% CI 1.56–4.31, *p* < 0.001). A nominally significant interaction was observed for age (P_interaction = 0.016). The dashed line indicates HR = 1.0. (B) Comparison of baseline characteristics between patients included in (*n* = 547) and excluded from (*n* = 154) the primary multivariable model, demonstrating comparable demographics and outcomes. *p* values calculated by chi‐squared test (categorical) or Mann–Whitney *U* test (continuous); no significant differences observed (all *p* > 0.05). (C) Landmark sensitivity analysis for FLC non‐normalization. Hazard ratios were estimated using Cox models adjusted for R‐ISS stage and age at landmark times of 3, 4, 5, and 6 months. The starting cohort consisted of 762 patients with C1‐C4 sFLC data and PFS follow‐up. The panel reports the number of patients and PFS events excluded before each landmark, the complete‐case Cox model sample size and event count, and the corresponding adjusted HRs. (D) Summary of prespecified sensitivity analyses. HRs ranged from 2.10 to 2.42 across eight formally tested analyses, including the original five (no landmark, C1–C8 window, exclusion of ASCT‐missing, restriction to baseline FLC‐abnormal, and adjustment for PI‐based therapy) and three measurement‐frequency analyses (≥ 2 sFLC measurements, adjustment for measurement count, and C4 measurement available). The S4 analysis (late normalizers vs. patients with no normalization through C8) is shown descriptively (log‐rank P only); a formal Cox comparison was not performed because this comparison is subject to immortal time bias when anchored at the 4‐month landmark.

The primary analysis evaluated FLC ratio status (within vs. outside the IMWG normal range) at any C1–C4 assessment, irrespective of baseline; this captures the combined prognostic effect of treatment‐induced normalization and baseline‐normal biology. A prespecified sensitivity analysis (S5) restricted to baseline‐abnormal patients isolated the treatment‐induced component. Baseline characteristics were comparable between patients included in (*n* = 547) and excluded from (*n* = 154) the primary multivariable model (Figure 4B).

After a median follow‐up of 33.7 months from the 4‐month landmark, 210 PFS events (38.4%) occurred among 547 patients with complete multivariable data. Kaplan‐Meier analysis showed significantly inferior PFS for FLC non‐normalized compared with FLC‐normalized patients (log‐rank *p* < 0.001; Figure 2A).

On univariable Cox regression, FLC non‐normalization was associated with significantly worse PFS (HR 2.06, 95% CI 1.57–2.70, *p* < 0.001). In the multivariable model adjusting for R‐ISS stage and age, FLC non‐normalization remained associated with inferior PFS (HR 2.10, 95% CI 1.60–2.76, *p* < 0.001; Figure 2C). R‐ISS stage was also a strong independent predictor (HR 2.19 per stage increment, 95% CI 1.76–2.72, *p* < 0.001), whereas age was not significant in the multivariable model (HR 1.01, *p* = 0.249). The addition of FLC normalization status to a base model containing R‐ISS stage and age improved the C‐index from 0.618 to 0.653 (delta = +0.035).

To isolate treatment‐induced normalization, the prespecified S5 analysis restricted to baseline‐abnormal patients (*n* = 250, 127 events) yielded HR 2.17 (95% CI 1.43–3.28, *p* < 0.001). A three‐group analysis showed a monotonic gradient with median PFS not reached for baseline‐normal patients (*n* = 257), 36.9 months for treatment‐induced normalization (*n* = 114), and 18.9 months for persistent abnormality (*n* = 198; Figure 1C).

In a response‐adjusted model (FLC + R‐ISS + age + best response, *n* = 527, 206 events), FLC non‐normalization remained independently associated with inferior PFS, although attenuated (HR 1.58, 95% CI 1.17–2.14, *p* = 0.003). In a fully adjusted model (*n* = 486, 189 events), the FLC HR was 1.69 (95% CI 1.22–2.35, *p* = 0.002; Figure 2D).

For OS (101 events), FLC non‐normalization was associated with inferior survival (multivariable HR 1.96, 95% CI 1.32–2.90, *p* < 0.001; Figure 2B). In a response‐adjusted OS model, the HR was attenuated and non‐significant (HR 1.33, *p* = 0.21), limited by modest event counts.

### 3.3 Normalization Timing and Subgroup Analyses

Among 433 patients who achieved FLC normalization, 355 (82.0%) normalized early (C1–C2) and 78 (18.0%) normalized mid‐course (C3–C4). The overall log‐rank test across the three groups was significant (*p* < 0.001; Figure 1A). Early and mid‐course normalizers did not differ significantly (*p* = 0.518), whereas both groups had better PFS than patients with no C1‐C4 normalization (*p* < 0.001 for early vs. no C1‐C4 normalization; *p* = 0.014 for mid‐course vs. no C1‐C4 normalization).

Forest plot analysis (Figure 4A) showed a consistent detrimental effect of FLC non‐normalization across all prespecified subgroups, with HRs ranging from 1.59 to 4.83. The direction of association was consistent across all evaluable subgroups, with several subgroups reaching statistical significance, including patients with high‐risk cytogenetics by FISH (HR 2.50, 95% CI 1.57–3.99, *p* < 0.001, *n* = 134). In the multivariable‐evaluable non‐transplant subgroup (*n* = 476, 198 events), FLC non‐normalization remained associated with inferior PFS (HR 2.29, 95% CI 1.72–3.04, *p* < 0.001). Consistent with this finding, Kaplan–Meier analysis of the broader non‐transplant population (*n* = 607; Figure 3B) showed similar separation between groups (median PFS 48.2 vs. 18.9 months, log‐rank *p* < 0.001).

A nominally significant interaction was observed between FLC normalization status and age (P_interaction = 0.016, uncorrected for multiple testing). In an age‐stratified multivariable Cox analysis adjusting for R‐ISS (*n* = 547), the HR for FLC non‐normalization was 2.86 (95% CI 1.92–4.26) in patients ≤ 65 years (*n* = 294) versus 1.59 (95% CI 1.08–2.34) in patients > 65 years (*n* = 253). In a sensitivity analysis restricted to 541 patients with available baseline eGFR data, additional adjustment for eGFR did not materially alter the HR in elderly patients (1.58 vs. 1.63 unadjusted; eGFR itself was not significant, *p* = 0.35; Figure 3D), indicating that the attenuated prognostic effect in the elderly is not explained by renal function differences. No significant interactions were observed for sex (*p* = 0.607), ISS stage (*p* = 0.683), R‐ISS stage (*p* = 0.268), FISH risk (*p* = 0.394), ASCT status (*p* = 0.305), or first‐line treatment (*p* = 0.725).

### 3.4 FLC and MRD Status and Sensitivity Analyses

MRD data during C1–C4 were available for 627 of 701 patients (89.4%). Among these, 244 (38.9%) achieved MRD negativity at any point during C1–C4. Within this MRD‐evaluable subset, 400 patients had achieved FLC normalization and 227 had not. FLC normalization was strongly associated with MRD negativity: the rate of ever MRD‐negativity was 51.2% (205/400) in FLC‐normalized patients versus 17.2% (39/227) in FLC non‐normalized patients (*p* < 0.001).

Among 495 patients with complete data for FLC, MRD, R‐ISS, and age (187 PFS events), failure to normalize was associated with a PFS HR of 2.42 (95% CI 1.80–3.25, *p* < 0.001) before MRD adjustment. In a multivariable model including both FLC and MRD status, FLC non‐normalization remained associated with inferior PFS (HR 1.94, 95% CI 1.42–2.65, *p* < 0.001), as did MRD negativity (HR 0.52, 95% CI 0.34–0.74, *p* < 0.001). For OS (87 events), FLC non‐normalization remained significant after MRD adjustment (HR 1.87, 95% CI 1.17–2.97, *p* = 0.01), whereas MRD negativity did not reach significance (HR 0.73, 95% CI 0.44–1.21, *p* = 0.22); limited OS events may have reduced power to detect an MRD effect. The combined model (FLC + MRD + R‐ISS + age) yielded the highest C‐index for PFS (0.676), compared with FLC alone (0.665), MRD alone (0.659), or the base model (R‐ISS + age, 0.621).

Kaplan–Meier analysis stratified by the four FLC/MRD combinations (Figure 3A) showed clear prognostic separation (log‐rank *p* < 0.001). Patients with both FLC normalization and MRD negativity had the best outcomes (median PFS 60.0 months), while those with neither had the worst (median PFS 18.9 months). Among MRD‐negative patients, PFS was favorable regardless of FLC status (FLC‐normalized: median 60.0 months; FLC non‐normalized: median not reached, though limited by small sample size [*n* = 39, 11 events]; pairwise *p* = 0.197). In contrast, among MRD‐positive patients, FLC normalization was associated with significantly better PFS (median 43.0 vs. 18.9 months, *p* < 0.001), suggesting that FLC status may provide additional prognostic stratification in the MRD‐positive population.

The primary finding was robust across the five formally tested sensitivity analyses (Figure 4D), with HRs ranging from 2.10 to 2.42. Among 206 patients classified as FLC non‐normalized by C4, 40 (19.4%) achieved normalization during C5‐C8 (“late normalizers”). A formal Cox comparison was not performed due to immortal time bias; descriptively, late normalizers had longer PFS than patients with no normalization through C8.

In an expanded landmark sensitivity analysis, the number of patients excluded before the 3‐, 4‐, 5‐, and 6‐month landmarks was 44, 61, 96, and 127, respectively. These included 30, 39, 59, and 79 pre‐landmark PFS events. The corresponding complete‐case Cox model populations were 562/219, 547/210, 518/193, and 496/181 patients/events. Despite these changes in the analytic population, the adjusted HR for FLC non‐normalization remained stable across landmark definitions: 2.19, 2.10, 2.08, and 2.04, respectively (Figure 4C). Across assessment windows C1‐C2 through C1‐C8, the HR was also stable (HR 2.03–2.42; Supporting Information S1: Figure S2).

A stricter sensitivity analysis used an operational definition of MRD‐negativity (sustained MRD‐negative: ≥ 2 consecutive negative MRD assessments during C1–C4 with the last assessment also negative; *n* = 101 sustained‐negative, 94 unstable, and 300 never‐negative). Under this definition, FLC non‐normalization remained associated with inferior PFS (HR 2.21, 95% CI 1.64–2.98, *p* < 0.001), and sustained MRD‐negativity was protective (HR 0.52, 95% CI 0.33–0.84, *p* = 0.007; Table S2). This pragmatic definition does not meet the IMWG criterion of ≥ 12‐month sustained MRD‐negativity, which is precluded by the C1–C4 induction window.

Measurement frequency did not differ between groups (median 4 assessments each, *p* = 0.33). The HR was unchanged after restricting to patients with ≥ 2 measurements (HR 2.15), restricting to those with an evaluable C4 (HR 2.16), or adjusting for measurement count (HR 2.10, with count itself non‐significant, *p* = 0.40; Figure 4D).

### 3.5 FLC and MRD Kinetics and Direct Comparison

In 279 patients with baseline‐abnormal FLC and at least one MRD assessment, the median time to documented FLC normalization was 7.0 versus 13.0 months for documented MRD negativity. FLC preceded MRD in 36 of 68 patients with both events, MRD preceded FLC in 13, and 19 were synchronous within 0.5 months. Cumulative normalization rates at 3, 6, and 12 months were 23.6%/44.9%/64.3% for FLC versus 9.9%/21.0%/41.8% for MRD (log‐rank *p* < 0.001; Figure 3E).

Because sFLC was assessed more frequently than MRD, we performed a paired‐visit‐restricted sensitivity analysis using only cycle visits with concurrent sFLC ratio and MRD results. In this stricter subset (*n* = 276), the median time to documented FLC normalization remained shorter than that to documented MRD negativity (6.8 vs. 13.0 months; log‐rank *p* < 0.001; Supporting Information S1: Figure S3). These findings suggest that earlier documentation of sFLC normalization was not solely explained by the higher frequency of sFLC testing, although the retrospective assessment schedule still warrants cautious interpretation.

At the C4 time point (*n* = 378), C4 FLC abnormality and C4 MRD positivity were similarly associated with PFS (FLC HR 2.05, *p* < 0.001; MRD HR 2.09, *p* < 0.001; Table S1). Under the ever C1–C4 definition (*n* = 495, primary analysis), FLC remained significantly associated with PFS (HR 2.19, 95% CI 1.61–2.97, *p* < 0.001), whereas the MRD association was attenuated (HR 1.55, *p* = 0.027). MRD has been reported to outperform FLC‐based response criteria, including stringent complete response, in prognostic discrimination among CR patients [24]. The FLC hazard ratio increased monotonically across four MRD definitions of varying stringency (range 1.85–2.21; Table S2).

OS associations differed between C4 and ever C1–C4 definitions. At C4, MRD positivity was the stronger predictor (HR 2.03, *p* = 0.026) while C4 FLC did not reach significance (HR 1.46, *p* = 0.177); under the ever C1–C4 definition, FLC was significant (HR 2.03, *p* = 0.002) while ever‐MRD was not (HR 1.15, *p* = 0.613). These OS comparisons rest on 65–87 events and should be regarded as exploratory.

### 3.6 Comparison With Established Prognostic Markers

In a matched subset of 266 patients (108 PFS events) with all six predictors available (Table S4), C4 FLC abnormality had the highest univariate hazard ratio (HR 2.91, 95% CI 1.99–4.27), followed by baseline FLC (HR 2.84), best response <VGPR (2.65), R‐ISS (2.26), FISH high‐risk (2.13), and C4 MRD positivity (2.10). C4 FLC also produced the largest C‐index gain over the R‐ISS plus age base model (+0.073), close to best response (+0.072) and above the other markers. In a full model with all six predictors, C4 FLC was borderline significant (HR 1.67, *p* = 0.051), as was C4 MRD (HR 1.63, *p* = 0.063); removing best response restored C4 FLC to *p* < 0.01.

## 4 Discussion

In this retrospective cohort study of 701 newly diagnosed MM patients from a single Chinese center, we observed that serum FLC ratio non‐normalization by cycle 4 was associated with inferior PFS and OS. After adjustment for R‐ISS stage and age using a landmark analysis approach, FLC non‐normalization remained associated with inferior PFS (HR 2.10, 95% CI 1.60–2.76) and OS (HR 1.96, 95% CI 1.32–2.90). The direction of association was consistent across all evaluable clinical subgroups, and the effect estimate remained stable across multiple sensitivity analyses.

The primary analysis reflects both treatment‐induced normalization and baseline‐normal biology, since the IMWG range is applied without reference to pre‐treatment values. The prespecified S5 analysis restricted to baseline‐abnormal patients (*n* = 250) yielded an essentially unchanged HR of 2.17, confirming that the prognostic effect is not driven by baseline biology alone.

Our HR of 2.10 aligns with the Tacchetti estimate (equivalent ∼2.17, *n* = 339) [13] and is somewhat higher than Klein's (∼1.64, *n* = 590) [18]. The difference likely reflects cohort characteristics (real‐world vs. trial; ASCT 12.6% vs. 100%) and analytic approach (landmark analysis vs. time‐dependent Cox regression).

The clinical utility of FLC normalization lies in its simplicity: unlike MRD, which requires bone marrow aspiration and specialized flow cytometry or sequencing, sFLC is a routine peripheral blood test. In the primary timing analysis, sFLC normalization was documented earlier than MRD negativity (median 7.0 vs. 13.0 months; Figure 3E). Because sFLC was monitored more frequently than MRD, this comparison is susceptible to ascertainment bias. However, in a paired‐visit‐restricted sensitivity analysis limited to cycle visits with concurrent sFLC and MRD results, the timing difference persisted (median 6.8 vs. 13.0 months; Supporting Information S1: Figure S3). Thus, sFLC may be useful for earlier serial monitoring, whereas MRD remains a deeper marrow‐based assessment. The two markers are best interpreted as complementary rather than interchangeable.

The 38.2% of patients classified as FLC non‐normalized by C4 represent a high‐risk population that might benefit from closer monitoring and prospective evaluation of response‐adapted strategies. Among 206 patients with C5–C8 data, 40 (19.4%) subsequently normalized and descriptively had longer PFS than patients with no normalization through C8, suggesting that FLC dynamics may retain prognostic information beyond cycle 4; a formal comparison was precluded by immortal time bias. Adding FLC to the R‐ISS + age base model improved discrimination modestly (C‐index 0.618–0.653), consistent with a complementary rather than transformative role.

Adjustment for MRD status attenuated but did not eliminate the FLC association (PFS HR 2.42 to 1.94; OS HR 1.87, *p* = 0.01), indicating partial overlap with complementary information.

The four‐group Kaplan–Meier analysis (Figure 3A) further clarified the nature of this complementarity. Among MRD‐negative patients, PFS was favorable regardless of FLC status (*p* = 0.197), although the FLC non‐normalized/MRD‐negative subgroup comprised only 39 patients with 11 events, limiting the reliability of this comparison. In contrast, among MRD‐positive patients (61.1% of evaluable), FLC normalization identified a subgroup with better outcomes (exploratory), with median PFS of 43.0 versus 18.9 months (*p* < 0.001). FLC normalization appeared to add prognostic value primarily within MRD‐positive patients, possibly reflecting ongoing clonal suppression beyond a single marrow assessment.

The FLC hazard ratio increased monotonically with stricter MRD definitions (1.85–2.21; Table S2), indicating that FLC and MRD capture partly distinct prognostic information. A direct comparison with established markers further supported C4 FLC's prognostic value (Table S4).

Notably, the choice of MRD detection platform may influence these findings: our NGF panel included kappa/lambda light chains, which may capture some of the same clonal information as the sFLC assay, yet FLC normalization still added complementary prognostic value. Higher‐sensitivity NGS‐based MRD at 10^−6^ sensitivity might further reduce concordance with FLC, a question for future studies [25].

A nominally significant age interaction (P_interaction = 0.016) showed a stronger effect in patients ≤ 65 years (HR 2.86) than > 65 years (HR 1.59; Figure 3D). The attenuation was not explained by eGFR; FLC remained significant in the elderly (*p* = 0.018). Possible explanations include competing mortality and age‐related biology.

We adopted a 4‐month landmark to mitigate immortal time bias [26], aligning with completion of four induction cycles. The 61 patients excluded before the landmark had higher‐risk baseline features and a higher rate of C1‐C4 FLC non‐normalization than the landmark cohort (Table S5), indicating potential selection toward landmark‐evaluable patients. Therefore, the primary estimates should be interpreted in this population rather than in all patients from diagnosis. However, HRs were stable across 3‐, 4‐, 5‐, and 6‐month landmarks, supporting that the association was not driven by the specific 4‐month landmark choice.

This study has several limitations. First, it was a single‐center retrospective study, and external validation in prospective cohorts is essential [27, 28, 29]. Second, R‐ISS data were missing in 22% of patients, although an ISS‐based model produced consistent results (Table S3). Third, MRD was assessed by NGF at 10‐5 sensitivity. Fourth, the predominantly non‐transplant population (ASCT 12.6%) limits generalizability to transplant‐eligible settings. Fifth, sFLC testing was performed using a single platform, and results may not generalize to alternative assays. Finally, although the paired‐visit‐restricted sensitivity analysis reduced measurement‐frequency bias, the retrospective and non‐uniform scheduling of sFLC and MRD assessments precludes definitive inference about true biological precedence between the two markers.

## 5 Conclusions

Early sFLC response during induction was associated with outcomes in newly diagnosed multiple myeloma and provided complementary prognostic information to established risk factors and MRD. Its incremental discriminative value was modest, but the marker is blood‐based, repeatable, and clinically accessible. Prospective validation is needed before using sFLC normalization as an early decision point in induction therapy.

## Author Contributions

Y.P. performed the analysis and wrote the manuscript. Y.W., Y.X., P.L. (Li), and C.Y. contributed to data collection. P.L. (Liu) supervised the study and revised the manuscript. All authors approved the final version.

## Funding

Science and Technology Innovation Action Plan of Shanghai (21YF1406300); Natural Science Foundation of Shanghai (22ZR1411400).

## Ethics Statement

The study was conducted in accordance with the principles of the Declaration of Helsinki and was approved by the Ethics Committee of Zhongshan Hospital, Fudan University (Approval No. B2017‐031R).

## Consent

Written informed consent was obtained from all patients prior to participation, permitting the use of their clinical and laboratory data for research.

## Conflicts of Interest

The authors declare no conflicts of interest.

## Supporting information


Supporting Information S1

Table S1

Table S2

Table S3

Table S4

Table S5

## Data Availability

The data that support the findings of this study are available from the corresponding author upon reasonable request.
