# Supplementary material for: Early Serum Free Light Chain Response in Newly Diagnosed Multiple Myeloma: Documented Earlier Than MRD and Complementary in Prognosis
Source: Hematol Oncol. 2026 Aug 4;44(5):e70234. doi: 10.1002/hon.70234 (PMC13435342; doi:10.1002/hon.70234)
Supplement: Supplementary file 1 [file HON-44-e70234-s001.docx]

**Sensitivity analysis of the combined FLC + MRD multivariable**

**Cox model under four alternative MRD definitions**

| **MRD definition** | **n** | **PFS events** | **FLC non-normalization**  **HR (95% CI)** | **FLC P** | **MRD HR**  **(95% CI)** | **MRD P** | **Model**  **C-index** |
| --- | --- | --- | --- | --- | --- | --- | --- |
| C4-only negative | 378 | 143 | 1.85 (1.30–2.64) | **<0.001** | 0.43 (0.28–0.64) | **<0.001** | 0.696 |
| Any negative C1–C4 (primary) | 495 | 187 | 1.94 (1.42–2.65) | **<0.001** | 0.52 (0.36–0.74) | **<0.001** | 0.676 |
| Best negative across C1–C8 | 501 | 188 | 2.05 (1.51–2.77) | **<0.001** | 0.59 (0.43–0.80) | **<0.001** | 0.676 |
| Sustained negative | 495 | 187 | 2.21 (1.64–2.98) | **<0.001** | 0.52 (0.33–0.84) | **0.007** | 0.669 |

All models: Cox(PFS_lm ~ FLC non-normalization + MRD definition + R-ISS + age), 4-month landmark. Definitions ordered by increasing stringency. C4-only: MRD-negative at C4 assessment only. Any negative C1–C4: MRD-negative at any assessment during C1–C4 (manuscript primary definition). Best negative C1–C8: MRD-negative at any assessment during C1–C8 (includes look-ahead). Sustained negative: ≥2 consecutive negative assessments during C1–C4 with the last assessment also negative. The FLC hazard ratio increases monotonically from 1.85 to 2.21 as the MRD definition becomes more stringent. Under the sustained-negative definition, three-group median PFS was: sustained negative (n=101), not reached; unstable (n=94), 60.0 months; no MRD negativity (n=300), 32.8 months. HR, hazard ratio; CI, confidence interval; PFS, progression-free survival.

**Caption supplied for production (matches the manuscript)**

Supplementary Table S2. Sensitivity analysis of the FLC + MRD + R-ISS + age Cox model under four MRD definitions, ordered by stringency. C4-only: negative at C4. Ever C1–C4 (primary): negative at any C1–C4. Best C1–C8: negative at any C1–C8. Sustained: ≥2 consecutive negatives during C1–C4 with the last also negative (median PFS: sustained 101 patients, not reached; unstable 94, 60.0 months; never negative 300, 32.8 months). The FLC hazard ratio remained significant and was somewhat higher under stricter MRD definitions (1.85 to 2.21). All analyses used a 4-month landmark.
