# Supplementary material for: Early Serum Free Light Chain Response in Newly Diagnosed Multiple Myeloma: Documented Earlier Than MRD and Complementary in Prognosis
Source: Hematol Oncol. 2026 Aug 4;44(5):e70234. doi: 10.1002/hon.70234 (PMC13435342; doi:10.1002/hon.70234)
Supplement: Supplementary file 2 [file HON-44-e70234-s002.docx]

**Direct comparison of C4 FLC and**

**established prognostic markers in**

**a common subset (n = 266)**

All analyses restricted to patients with non-missing data on all six predictors and PFS as outcome with 4-month landmark.

**Univariate Cox in common subset**

| **Predictor** | **HR (95% CI)** | **P** | **C-index** |
| --- | --- | --- | --- |
| C4 FLC abnormality | 2.91 (1.99–4.27) | **<0.001** | 0.649 |
| Baseline FLC abnormality | 2.84 (1.83–4.42) | **<0.001** | 0.610 |
| Best response <VGPR | 2.65 (1.81–3.89) | **<0.001** | 0.636 |
| R-ISS (per stage) | 2.26 (1.68–3.04) | **<0.001** | 0.637 |
| FISH high-risk | 2.13 (1.44–3.16) | **<0.001** | 0.587 |
| C4 MRD positive | 2.10 (1.33–3.31) | **0.001** | 0.586 |

**Full multivariable Cox model (all predictors simultaneously, C-index = 0.742)**

| **Variable** | **HR (95% CI)** | **P** |
| --- | --- | --- |
| R-ISS (per stage) | 2.65 (1.82–3.85) | **<0.001** |
| Best response <VGPR | 2.21 (1.46–3.34) | **<0.001** |
| C4 FLC abnormality | 1.67 (1.00–2.81) | 0.051 |
| C4 MRD positive | 1.63 (0.97–2.74) | 0.063 |
| Baseline FLC abnormality | 1.49 (0.86–2.61) | 0.157 |
| FISH high-risk | 1.10 (0.70–1.73) | 0.680 |
| Age (per year) | 0.99 (0.97–1.02) | 0.569 |

**Incremental C-index over R-ISS + age (base C-index = 0.634)**

| **Added predictor** | **C-index (with predictor)** | **ΔC** |
| --- | --- | --- |
| C4 FLC abnormality | 0.707 | +0.073 |
| Best response <VGPR | 0.706 | +0.072 |
| Baseline FLC abnormality | 0.680 | +0.046 |
| C4 MRD positive | 0.675 | +0.041 |
| FISH high-risk | 0.636 | +0.002 |

Common subset: n = 266, 108 PFS events, with non-missing data on all six predictors plus age. C4 FLC abnormality: FLC κ/λ ratio outside 0.26–1.65 at C4 assessment. Best response is subject to look-ahead bias (ascertained over the entire first-line course). The borderline P value for C4 FLC in the full multivariable model reflects partial collinearity with best response and baseline FLC; when best response is excluded, C4 FLC P < 0.01. 4-month landmark; HR, hazard ratio; CI, confidence interval; ΔC, change in C-index over the R-ISS + age base model (C-index 0.634).

**Caption supplied for production (matches the manuscript)**

Supplementary Table S4. Direct comparison of C4 FLC and established prognostic markers in a common subset (n = 266, 108 PFS events) with non-missing data on all six predictors plus age (C4 FLC, R-ISS, C4 MRD, IMWG best response, FISH risk, baseline FLC, and age). Three subtables: (1) univariate Cox with HR and C-index for each predictor; (2) full multivariable Cox model with all six predictors plus age (C-index 0.742); (3) incremental C-index over the R-ISS plus age base model. C4 FLC abnormality ranked first by univariate HR (2.91) and ΔC-index (+0.073). In the full multivariable model, C4 FLC was borderline significant (HR 1.67, P = 0.051), reflecting collinearity with best response. 4-month landmark; PFS as outcome.
