# Supplementary material for: Early Serum Free Light Chain Response in Newly Diagnosed Multiple Myeloma: Documented Earlier Than MRD and Complementary in Prognosis
Source: Hematol Oncol. 2026 Aug 4;44(5):e70234. doi: 10.1002/hon.70234 (PMC13435342; doi:10.1002/hon.70234)
Supplement: Supplementary file 3 [file HON-44-e70234-s003.docx]

| **Characteristic** | **Excluded before 4-mo landmark (n=61)** | **Included landmark cohort (n=701)** | **P value** |
| --- | --- | --- | --- |
| Age, years, median (IQR) | 64.0 (56.0-69.0) | 64.0 (57.0-71.0) | 0.782 |
| Male, n (%) | 42/61 (68.9) | 440/701 (62.8) | 0.344 |
| **ISS stage, n (%)** |  |  | **0.034** |
| 1 | 10 (16.4) | 200 (28.6) |  |
| 2 | 15 (24.6) | 201 (28.7) |  |
| 3 | 36 (59.0) | 299 (42.7) |  |
| **R-ISS stage, n (%)** |  |  | **0.002** |
| 1 | 4 (7.7) | 125 (22.9) |  |
| 2 | 29 (55.8) | 319 (58.3) |  |
| 3 | 19 (36.5) | 103 (18.8) |  |
| High-risk FISH, n (%) | 20/55 (36.4) | 152/608 (25.0) | 0.066 |
| Baseline FLC ratio, median (IQR) | 0.27 (0.04-1.10) | 0.87 (0.27-2.29) | **0.008** |
| Baseline FLC ratio abnormal, n (%) | 33/47 (70.2) | 312/569 (54.8) | **0.041** |
| **C1-C4 FLC normalization status, n (%)** |  |  | **<0.001** |
| FLC-normalized | 19/61 (31.1) | 433/701 (61.8) |  |
| FLC non-normalized | 42/61 (68.9) | 268/701 (38.2) |  |
| ASCT received, n (%) | 0/59 (0.0) | 88/695 (12.7) | **0.001** |
| **First-line treatment, n (%)** |  |  | **0.005** |
| PI+IMiD | 21 (34.4) | 339 (48.4) |  |
| PI-only | 24 (39.3) | 253 (36.1) |  |
| IMiD-only | 0 (0.0) | 24 (3.4) |  |
| Other | 16 (26.2) | 85 (12.1) |  |
| PFS follow-up, months, median (IQR) | 2.3 (1.4-3.1) | 20.7 (10.6-38.8) | **<0.001** |
| PFS event, n (%) | 39/61 (63.9) | 275/701 (39.2) | **<0.001** |

Abbreviations: FLC, free light chain; FISH, fluorescence in situ hybridization; ASCT, autologous stem cell transplantation; PI, proteasome inhibitor; IMiD, immunomodulatory drug; IQR, interquartile range.

Statistics: Continuous variables were compared by Mann–Whitney U test; categorical variables by chi-square or Fisher's exact test. Bold P values indicate P < 0.05. The excluded group included 39 pre-landmark PFS events and 22 patients with PFS follow-up not extending beyond 4 months.

**Caption supplied for production (matches the manuscript)**

Supplementary Table S5. Baseline and early FLC characteristics of patients excluded before the 4-month landmark compared with the landmark analysis cohort.
