# Supplementary material for: Early Serum Free Light Chain Response in Newly Diagnosed Multiple Myeloma: Documented Earlier Than MRD and Complementary in Prognosis
Source: Hematol Oncol. 2026 Aug 4;44(5):e70234. doi: 10.1002/hon.70234 (PMC13435342; doi:10.1002/hon.70234)
Supplement: Supplementary file 4 [file HON-44-e70234-s004.docx]

**Direct comparison of FLC and MRD as PFS/OS predictors in multivariable Cox models**

| **Model** | **Variable** | **HR** | **95% CI** | **P value** |
| --- | --- | --- | --- | --- |
| **C4 time-point model (PFS)** | n=378, events=143, C-index=0.705 |  |  |  |
|  | C4 FLC abnormality | 2.05 | 1.42-2.96 | **<0.001** |
|  | C4 MRD positivity | 2.09 | 1.36-3.22 | **<0.001** |
|  | R-ISS stage (per stage) | 2.81 | 2.13-3.72 | **<0.001** |
|  | Age (per year) | 1.00 | 0.98-1.02 | 0.846 |
| **C4 time-point model (OS)** | n=378, events=65, C-index=0.702 |  |  |  |
|  | C4 FLC abnormality | 1.46 | 0.84-2.51 | 0.177 |
|  | C4 MRD positivity | 2.03 | 1.09-3.79 | **0.026** |
|  | R-ISS stage (per stage) | 2.85 | 1.89-4.31 | **<0.001** |
|  | Age (per year) | 1.02 | 0.99-1.05 | 0.302 |
| **C1–C4 status model (PFS)** | n=495, events=187, C-index=0.665 |  |  |  |
|  | FLC non-normalization (C1–C4) | 2.19 | 1.61-2.97 | **<0.001** |
|  | MRD positivity (C1–C4) | 1.55 | 1.05-2.27 | **0.027** |
|  | R-ISS stage (per stage) | 2.44 | 1.92-3.09 | **<0.001** |
|  | Age (per year) | 1.00 | 0.99-1.02 | 0.770 |
| **C1–C4 status model (OS)** | n=495, events=87, C-index=0.679 |  |  |  |
|  | FLC non-normalization (C1–C4) | 2.03 | 1.30-3.17 | **0.002** |
|  | MRD positivity (C1–C4) | 1.15 | 0.67-1.96 | 0.613 |
|  | R-ISS stage (per stage) | 2.49 | 1.76-3.53 | **<0.001** |
|  | Age (per year) | 1.01 | 0.99-1.04 | 0.260 |

Bold p values indicate statistical significance (P < 0.05).

**Caption supplied for production (matches the manuscript)**

Supplementary Table S1. Direct comparison of FLC and MRD as PFS and OS predictors in multivariable Cox models. Two models contrast C4 time-point status (n = 378) with ever-status across C1–C4 (n = 495). All models use a 4-month landmark and adjust for R-ISS stage and age.
