# Supplementary material for: Early Serum Free Light Chain Response in Newly Diagnosed Multiple Myeloma: Documented Earlier Than MRD and Complementary in Prognosis
Source: Hematol Oncol. 2026 Aug 4;44(5):e70234. doi: 10.1002/hon.70234 (PMC13435342; doi:10.1002/hon.70234)
Supplement: Supplementary file 5 [file HON-44-e70234-s005.docx]

Supplementary Figure S1. Patient selection flow diagram. From the PCD database (n = 2,040), sequential exclusions yielded the analysis cohort (n = 701: FLC-normalized, 433; FLC non-normalized, 268), the primary multivariable model (n = 547), and the exploratory MRD analysis (n = 495).

Supplementary Figure S2. FLC non-normalization HR by assessment window. Hazard ratios for FLC non-normalization across assessment windows from C1–C2 through C1–C8 (HR range 2.03–2.42). The primary C1–C4 window (red diamond) yielded HR 2.10 (95% CI 1.60–2.76). Wider windows may inflate HR due to potential differential follow-up bias.

Supplementary Figure S3. Paired-visit-restricted sensitivity analysis of documented sFLC normalization and MRD negativity. The analysis included 276 patients with baseline-abnormal FLC and at least one cycle visit with concurrent sFLC ratio and MRD results. Both events were evaluated only at paired visits. Patients without documented normalization or MRD negativity were censored at the last paired visit. Median time to documented sFLC normalization was 6.8 months versus 13.0 months for documented MRD negativity (log-rank P < 0.001).
