# Supplementary material for: Early Serum Free Light Chain Response in Newly Diagnosed Multiple Myeloma: Documented Earlier Than MRD and Complementary in Prognosis
Source: Hematol Oncol. 2026 Aug 4;44(5):e70234. doi: 10.1002/hon.70234 (PMC13435342; doi:10.1002/hon.70234)
Supplement: Supplementary file 6 [file HON-44-e70234-s006.docx]

**ISS-based multivariable Cox model**

| **Model** | **Variable** | **HR** | **95% CI** | **P value** |
| --- | --- | --- | --- | --- |
| **PFS model** | n=700, events=274, C-index=0.633 |  |  |  |
|  | FLC non-normalization | 1.83 | 1.44-2.32 | **<0.001** |
|  | ISS | 1.56 | 1.34-1.82 | **<0.001** |
|  | Age | 1.01 | 1.00-1.02 | 0.101 |
| **OS model** | n=700, events=132, C-index=0.655 |  |  |  |
|  | FLC non-normalization | 1.53 | 1.09-2.16 | **0.015** |
|  | ISS | 1.92 | 1.52-2.43 | **<0.001** |
|  | Age | 1.01 | 0.99-1.03 | 0.292 |

Abbreviations: PFS, progression-free survival; OS, overall survival; FLC, free light chain; ISS, International Staging System; HR, hazard ratio; CI, confidence interval; C-index, concordance index.

**Bold P values indicate statistical significance (P < 0.05).**

**Caption supplied for production (matches the manuscript)**

Supplementary Table S3. ISS-based multivariable Cox model in the larger sample including patients without R-ISS data, demonstrating consistency of the FLC effect when staging information is operationalized via ISS rather than R-ISS (n = 700; PFS HR 1.83, 95% CI 1.44–2.32, P < 0.001).
